# What type of Bt corn is suitable for a region with diverse lepidopteran pests: A laboratory evaluation

**DOI:** 10.1080/21645698.2020.1831728

**Published:** 2020-10-21

**Authors:** Guoping Li, Hongqiang Feng, Tingjie Ji, Jianrong Huang, Caihong Tian

**Affiliations:** Henan Key Laboratory of Crop Pest Control, Key Laboratory of Crop Integrated Pest Management in Southern Region of North China, International Joint Research Laboratory for Crop Protection of Henan, Biological Pesticides Engineering Research Center of Henan Province, Institute of Plant Protection, Henan Academy of Agricultural Sciences, Zhengzhou, Henan, China

**Keywords:** Bt toxin, corn pests, patterns of susceptibility, bioassay

## Abstract

Transgenic crops that produce *Bacillus thuringiensis* (Bt) toxins are effective tools for controlling lepidopteran pests. However, the degree of susceptibility to Bt toxins differs among various pest species due to relatively narrow spectrum and high selectivity of such toxins. Bt corn hybrids for Chinese market were designed to target Asian corn borer *Ostrinia furnacalis* (Guenée), while their efficacy against other lepidopteran pests are not well defined, such as *Conogethes punctiferalis* (Guenée), *Helicoverpa armigera* (Hübner), *Agrotis ypsilon* (Rottemberg), and *Mythimna separata* (Walker), which are also important lepidopteran pests on corn in the Huang-Huai-Hai Summer Corn Region of China. To determine what type of Bt corn is suitable for this region, the efficacy of five Bt toxins, i.e., Cry1Ab, Cry1Ac, Cry1F, Cry2Ab, and Vip3A, to these five lepidopteran species was evaluated in laboratory. Both *O. furnacalis* and *C. punctiferalis* showed similar high susceptibility to all five Bt toxins. *A. ypsilon* and *M. separate* were less sensitive to Cry1Ab and Cry1Ac than the other species. *H. armigera, A. ypsilon* and *M. separate* were less sensitive to Cry1F than *O. furnacalis* and *C. punctiferalis. H. armigera* was more sensitive to Cry2Ab than other tested species. All five species were equally sensitive to Vip3A, though their LC_50_s were all relatively higher. These findings suggest that the first generation Bt corn expressing single Cry1 toxin should not be the first choice because of the potential risk of control failure or less efficacy against *H. armigera, A. ypsilon* or *M. separate*. The second-generation Bt corn expressing Cry1 and Cry2 toxins, or the third generation Bt corn expressing Cry1, Cry2 and Vip3A toxins might produce better protection of corn in the Huang-Huai-Hai Summer Corn Region of China.

## Introdction

Transgenic Bt (*Bacillus thuringiensis*) crops expressing specific insecticidal proteins are a great success for controlling major agricultural insect pests with a total acreage of 191.7 million ha in 2018, increased ~113-fold since its first commercialization in 1996.^1^ Area-wide benefits have been created by adaptation of transgenic crops through both yield increase and reduction of chemical pesticide sprays, especially during seasons of moderate to severe insect pest infestations.^2–4^ Additional gain of 186.1 billion US dollars in farmer income was generated by Bt crops during the past two decades.^1^

The first Bt corn expressing Cry1Ab toxin was commercially planted in 1996 in the United States to control the European corn borer, *Ostrinia nubilalis* (Hübner), one of the most destructive pests in the U.S. corn belt.^5^ In 2017, a total of 29.44 million ha of Bt corn was planted in U.S. with an adoption rate of 81.26%.^1^ Among many events, Cry1Ab, Cry1Ac, and Cry1F are usually expressed in single or pyramided Bt corns, while Cry2Ab and Vip3A are available only as stacked traits. Plants containing two different Bt proteins with dissimilar binding sites have a broader efficacy spectrum and a potential to delay pest resistance development more effectively than single Bt crops, thus are widely adopted in recent years.^6,7^ In addition to the U.S., Brazil, Argentina, Canada, South Africa, Uruguay, Spain, Honduras, Chile, Egypt, Romania, Slovakia, Czech Republic, Portugal, and Philippines also planted large areas of Bt corn by 2018.^1^

Unlike traditional broad-spectrum chemical insecticides, Bt has selective toxicity for a certain group of insects, i.e., mainly lepidopterans, and the toxicity level can vary greatly between different species. For example, the Cry1Ab corn in Europe exhibits excellent efficacy against the two key lepidopteran pests, *O. nubilalis* and the Mediterranean corn borer *Sesamia nonagrioides* Lefèbvre (Lepidoptera: Noctuidae),^8,9^ but has a much lower efficacy against the other two secondary lepidopteran pests, i.e., the true armyworm *Mythimna unipuncta* Haworth and the corn earworm *Helicoverpa armigera* (Hübner) (Lepidoptera: Noctuidae).^10,11^ In Canada, the Cry1Ab corn has effectively controlled *O. nubilalis* and other lepidopteran pests, such as the fall armyworm *Spodoptera frugiperda* (Smith), and the corn earworm *H. zea* (Boddie), but showed limited ability of controlling the western bean cutworm *Striacosta albicosta* (Smith).^12,13^ Thus, planting Bt crops has a potential risk of secondary insect pests becoming key pests in such scenario.^14^

Although Bt corn has not been commercialized in China yet, it is a promising technology to be considered for corn pest control in future. There are 7 corn production regions in China. They are Northeast Spring Corn Region (NeSCR), North Spring Corn Region (NSCR), Huang-Huai-Hai Summer Corn Region (HHHSCR), Southeast Hilly Corn Region (SeHCR), Southwest Hilly Corn Region (SwHCR), Northwest Inland Corn Region (NwICR), and Qing-Zang Plateau Corn Region (QZPCR).^15^ The HHHSCR is the largest region and accounts for up to 40% of the total corn cultivation acreage in China, followed by the NeSCR, the second largest region which accounts for 25% of the total acreage. The pest profiles of these production regions are different. In the NeSCR, Asian corn borer *Ostrinia furnacalis* (Guenée) is the only major pest species on corn, but in the HHHSCR, additional species including the yellow peach moth *Conogethes punctiferalis* (Guenée), *H. armigera* (Hübner), black cutworm *Agrotis ypsilon* (Rottemberg), and oriental armyworm *Mythimna separata* (Walker) are also considered as major lepidopteran pests of corn.^16^ Therefore, the efficacy of different types of Bt corns against various major corn pests should be characterized before commercialization.

*O. furnacalis*, a sibling species of *O. nubilalis*, is the most destructive corn pest in China. The larvae feed on leaves, silks, tassels, and also bore into cornstalks and ears, which can cause approximately 10% grain yield losses.^17–20^ In addition, infested ears aggravate the occurrence of corn ear rot and dramatically decrease the grain quality. Previous studies showed that *O. nubilalis* and *O. furnacalis* had similar sensitivity to Cry1Aa, Cry1Ab, Cry1Ac, Cry1Ba, and Cry1F toxins.^21^ In addition to laboratory bioassays, field experiments also showed that *O. furnacalis* was susceptible to Bt corn and Bt cotton expressing Cry1Ab or Cry1Ac.^22–25^ These results are useful references for regulatory decisions regarding the use of transgenic corn in areas where *O. furnacalis* is a major pest in China. However, information on the susceptibility of other major insect pests of corn to Bt toxins is limited.

*C. punctiferalis*, is a polyphagous pest damaging multiple crops, such as peach, chestnut, sunflower, sorghum, and corn.^26–28^ Similar to *O. nubilalis, C. punctiferalis* larvae mainly feed on silks and then bore into ears and tunnel inside, causing fungal infections and ear contamination in addition to the physical damage. In recent years, this pest has increased its population abundance and becomes a major pest on corn in HHHSCR, and its damage is even greater than that of *O. nubilalis* in some locations within the region.^16,29–31^

*H. armigera* is an omnivorous pest causing serious damage to not only cotton but also wheat, sorghum, tobacco, pepper, and specifically the reproductive organs of corn. Since corn is the major summer crop in HHHSCR, the fourth generation of *H. armigera* would feed mainly on this crop and has become a serious pest.^32,33^ The larvae of *H. armigera* concentrate at the top of ears feeding on silks during its early stages, thus affecting pollination. They also bore into grains resulting in extensive accumulations of feces and mildew, which can easily contaminate grains, and thus seriously reduce the grain quality.^32^

*A. ypsilon* is one of the most important underground pests. The larvae hide in the soil and feed on the stems of corn seedlings, which results in damaged growth points and plant death.^34^ This pest is very difficult to control by spraying chemical insecticides due to high resistance and its nocturnal feeding behavior.^35^

*M. separate* is a well-known long-distance migrant pest of many crops and is widely distributed throughout northern and southern China.^36,37^ It is a sporadic pest of corn, wheat, sugarcane, and other crops. Because of polyphagous, migratory, and sporadic nature of this pest, sudden outbreaks often occur in some areas, consuming all available leaves and causing serious yield losses.

In the present study, laboratory bioassays were conducted to evaluate the efficacy of Cry1Ab, Cry1Ac, Cry1F, Cry2Ab, and Vip3A toxins against *O. furnacalis, C. punctiferalis, H. armigera, A. ypsilon*, and *M. separate*, thus to provide technical suggestions for commercialization of Bt corn with specific focus on the HHHSCR of China.

## Materials and Methods

### Insects

A Bt-susceptible colony of *O. furnacalis* was established from a collection of approximately 200 adults trapped by a 1000 W search light trap during late May and early June 2015 in Yuanyang County of Henan province (35.13°N, 113.41°E). The eggs were collected using wax paper which was changed daily in a large adult rearing box (40 × 30 × 25 cm^3^). The hatched larvae were reared using a modified artificial diet based on the ingredients described in Song et al.^38^

A Bt-susceptible strain of *C. punctiferalis* was established from approximately 300 larvae collected from sorghum fields during late September 2016 in Yuanyang County. The initial 12 generations of this colony were kept on corn seedlings with chestnuts as oviposition substitute, and then switched to artificial diet with major ingredients of chestnut powder, wheat germ and soybean powder (unpublished data).

A Bt-susceptible *H. armigera* colony maintained in the laboratory was established from a field collection of approximately 200 larvae from non-Bt corn in Yuanyang County in 2016. The larvae were reared for over 20 generations using artificial diet based on corn and soybean powder as described in Liang et al.^39^

A Bt-susceptible laboratory population of *A. ypsilon* was established from a field collection of more than 200 moths from Yuanyang County during May 2015. A meridic diet specific to *A. ypsilon* was used for larval rearing.^40^

A Bt-susceptible laboratory population of *M. separata* was established from a field collection of more than 300 larvae from non-Bt corn in Lingbao County of Henan province (34.61°N, 110.80°E) in 2016. A meridic diet specific to *M. separata* was used for larval rearing.^41^

All adults were maintained with 10% sugar solution, and all colonies were reared under laboratory conditions with a temperature of 25–28°C, relative humidity of 60–80%, and photoperiod of L:D of 16:8 h. All colonies had been maintained in the absence of Bt selection for at least 1–2 years before testing. It should be noted that Bt corn has not been commercially planted in China except for very few rigorously supervised experimental plots. Corn pest control has heavily relied on spaying chemical pesticides with little use of Bt formulations in the regions where collections were made to establish the colonies of the five pest species used in this study. Although Cry1Ac transgenic cotton, the major host of *H. armigera*, has been widely planted in this region, the acreage is shrinking and the field population of *H. armigera* remains very susceptible to Cry1Ac.^42^ Hence, potential selection for Bt resistance alleles is very weak and susceptibility data of Bt toxins collected on these species should represent the baseline level.

### Bt Protein Preparation

Cry1Ab, Cry1Ac, and Cry1F 98% purified proteins were produced and provided by Marianne P. Carey, Case Western Reserve University (Ohio, U.S.). Bioassay solutions of these proteins were prepared by dissolving them in 50 mM 3-(cyclohexylamino) propanesulfonic acid (CAPS), pH 10.5 buffer. Cry2Ab and Vip3A were purchased from Beijing General Pest Biotech Research Co. Ltd. (Beijing, China). The Cry2Ab protein sample was delivered as a purified crystal solution of 1.5 mg/ml concentration. The protein crystals were solubilized by incubation in 0.1 M Na_2_CO_3_, pH 10.5. The purified Vip3A protein was delivered as a dry powder and freshly dissolved in distilled water before bioassay. All protein samples were stored in a freezer at −80°C.

### Bioassays

All bioassays were conducted during April 2017 to July 2018 using one testing procedure (diet-overlay). Approximately, 1.5 ml of liquefied diet was poured into each well (13 mm in depth and 16 mm in diameter, 2 cm^2^ surface area) of a 24-well plate. After the diet solidified, 40 µl of Bt protein solutions of seven to nine concentrations or control (distilled water and buffer contains 0.1% (v/v) Triton X-100) was added onto the diet surface in each well using a pipette. When all wells on a plate were treated, the plate was tilted from front to back and from left to right to ensure that the protein solution was evenly distributed on the diet surface. After the diet surface dried, one neonate (0 to 24-h-old) was transferred to each well with a fine brush (24 neonates per concentration). After infestation, the plate was covered with a sheet of blow-molded paper pad, then fastened with 2 rubber bands to prevent escape. The infested plates were placed in a climatic chamber at 27 ± 1°C, L:D of 16:8 h, and RH of 60 ± 10%. The bioassays were repeated three times for each species and each toxin. The sample size (total numbers of neonates) are listed in Table 1. Mortality was recorded at 7 days after treatment. Larvae that remained as ﬁrst instar throughout the experiment were counted as dead.

**Table 1.** Lethal concentrations of five *Bacillus thuringiensis* toxins to *Ostrinia furnacalis, Conogethes punctiferalis, Agrotis ypsilon, Mythimna separata, Helicoverpa armigera.*

Insect species	Bt Toxin	n^a^	Slope(±SE)	LC_50_(95%FL)^b^	LC_90_(95%FL)^b^	χ^2c^	df
*Ostrinia furnacalis*	Cry1Ab	432	1.62 ± 0.17	2.11(1.64–2.62)	13.06(9.53–20.38)	7.77	4
	Cry1Ac	432	1.64 ± 0.19	1.70(1.25–2.19)	10.29(7.49–16.26)	7.27	5
	Cry1F	432	1.43 ± 0.19	4.61(3.48–6.04)	36.52(22.67–78.80)	9.13	4
	Cry2Ab	504	1.39 ± 0.14	134.87(104.77–171.34)	1127.83(762.33–1957.88)	10.62	5
	Vip3A	576	0.51 ± 0.09	328.44(183.99–660.54)	104243.05(20067.75–2669993.94)	9.27	6
*Conogethes punctiferalis*	Cry1Ab	432	1.09 ± 0.16	3.41(2.20–4.96)	50.62(27.56–137.89)	8.54	4
	Cry1Ac	504	1.27 ± 0.11	8.62(6.90–11.02)	87.43(55.88–161.90)	8.27	5
	Cry1F	504	1.25 ± 0.17	10.03(6.79–14.26)	107.17(62.39–251.50)	3.93	5
	Cry2Ab	504	1.12 ± 0.16	126.70(83.81–180.59)	1762.68(961.45–4919.07)	10.92	5
	Vip3A	576	0.95 ± 0.10	406.36(291.52–584.47)	9158.11(4643.12–25184.36)	6.48	6
*Helicoverpa armigera*	Cry1Ab	432	1.24 ± 0.16	3.52(2.62–4.66)	48.27(22.85–223.72)	9.36	4
	Cry1Ac	504	1.07 ± 0.14	7.33(5.34–10.12)	191.10(86.45–753.18)	8.56	5
	Cry1F	576	1.40 ± 0.12	1513.22(1206.02–1887.17)	12497.37(8724.82–20238.11)	9.47	7
	Cry2Ab	360	1.04 ± 0.14	70.39(39.47–106.65)	1210.81(724.61–2678.95)	9.51	6
	Vip3A	576	0.98 ± 0.11	468.07(333.76–649.50)	9619.37(5322.10–2295.92)	11.17	6
*Agrotis ypsilon*	Cry1Ab	480	1.84 ± 0.34	762.61(530.76–1006.73)	3779.38(2477.67–8473.58)	10.73	6
	Cry1Ac	480	1.33 ± 0.12	1090.42(855.09–1381.42)	10022.41(7194.69–82230.30)	11.87	6
	Cry1F	720	0.68 ± 0.07	1027.84(695.06–1544.08)	79361.96(33296.01–290783.82)	12.66	8
	Cry2Ab	420	1.348 ± 0.13	367.38(279.49–466.63)	3352.74(2337.07–5521.13)	10.83	5
	Vip3A	504	1.32 ± 0.14	352.81(273.79–453.77)	3287.45(2095.47–6375.52)	8.23	5
*Mythimna separata*	Cry1Ab	432	1.01 ± 0.15	119.24(82.90–164.50)	2250.03(1152.64–7167.31)	5.36	4
	Cry1Ac	480	0.61 ± 0.10	132.25(67.50–217.81)	16821.22(5669.80–128957.09)	9.04	6
	Cry1F	624	0.84 ± 0.09	812.18(564.24–1141.42)	27304.07(14350.18–69978.33)	8.74	7
	Cry2Ab	432	1.57 ± 0.18	379.21(293.81–480.88)	2487.17(1691.91–4415.81)	4.73	4
	Vip3A	480	0.98 ± 0.11	336.61(237.57–467.64)	6744.22(3812.70–15452.58)	6.18	6

^a^total number of larvae tested in the bioassay.

^b^ng of Bt toxin/cm^2^ of treated artificial diet surface with 95% fiducial limits in parentheses.

^c^Chi-square goodness-of-fit test indicates all probit models were good fit (*P* > 0.05).

### Data Analysis

The mortalities of each species and each toxin showed no significant difference among the three repeats, thus the data were pooled for probit analysis to estimate the median lethal concentration (LC_50_) and the slope of the regression using Polo-plus progra.^43^ The goodness of fit was tested with Chi-square test. The comparisons of the LC_50_s (susceptibility) were based on their 95% fiducial limits. Significant difference was declared when the 95% fiducial limits was not overlapping. To compare the pattern of relative tolerance of each insect species to different Bt toxins, a tolerance ratio (TR) was calculated using the LC_50_s of *O. furnacalis* as the reference (denominator) for each type of Bt toxin.

## Results

### Susceptibility of Five Insect Species to Five Bt Toxins

The probit analysis results of *O. furnacalis, C. punctiferalis, H. armigera, A. ypsilon*, and *M. separate* for each Bt toxin are shown in Table 1. Chi-square tests indicated that all probit models were good fit for the mortality data (Table 1). For a given toxin, significant differences were present among the tested species. The LC_50_ values of Cry1Ac, Cry1Ab, and Cry1F for *O. furnacalis* were 1.70, 2.11, and 4.61 ng/cm^2^, respectively. This indicated that *O. furnacalis* was highly susceptible to these three Cry1 toxins. In contrast, the LC_50_s of Cry2Ab and Vip3A for *O. furnacalis* were 134.87 and 328.44 ng/cm^2^, respectively, which indicated that *O. furnacalis* was more tolerant to Cry2Ab and Vip3A than to Cry1 toxins. Similarly, *C. punctiferalis* was highly susceptible to the three Cry1 toxins but more tolerant to Cry2Ab and Vip3A. *H. armigera* had a different trend. It was highly susceptible to Cry1Ab (LC_50_ = 3.52 ng/cm^2^) and Cry1Ac (LC_50_ = 7.33 ng/cm^2^), but not to Cry1F (LC_50_ = 1513.22 ng/cm^2^). Additionally, it was relative more susceptible to Cry2Ab (LC_50_ = 70.39 ng/cm^2^) compared to Cry1F. *A. ypsilon* was not susceptible to the three Cry1 toxins with LC_50_ values ranged from 762.61 to 1090.42 ng/cm^2^, while *M. separate* was moderately susceptible to Cry1Ab, Cry1Ac, Cry2Ab, and Vip3A with LC_50_ values ranging from 119.24 to 379.21 ng/cm^2^.

### Patterns of Susceptibility

Based on the LC_50_ values and 95% fiducial limits (Table 1), the order of susceptibility to Cry1Ab was as follows: *O. furnacalis, C. punctiferalis, H. armigera* > *M.separata* > *A. ypsilon*; the order of susceptibility to Cry1Ac: *O. furnacalis* > *C. punctiferalis, H. armigera* > *M. separata* > *A. ypsilon*; the order of susceptibility to Cry1F: *O. furnacalis* > *C. punctiferalis* > *M. separata, A. ypsilon* > *H. armigera*; and the order of susceptibility to Cry2Ab: *O. furnacalis, C. punctiferalis, H. armigera* > *M. separata, A. ypsilon*. The susceptibility to Vip3A was similar among the five species.

**Figure 1.**
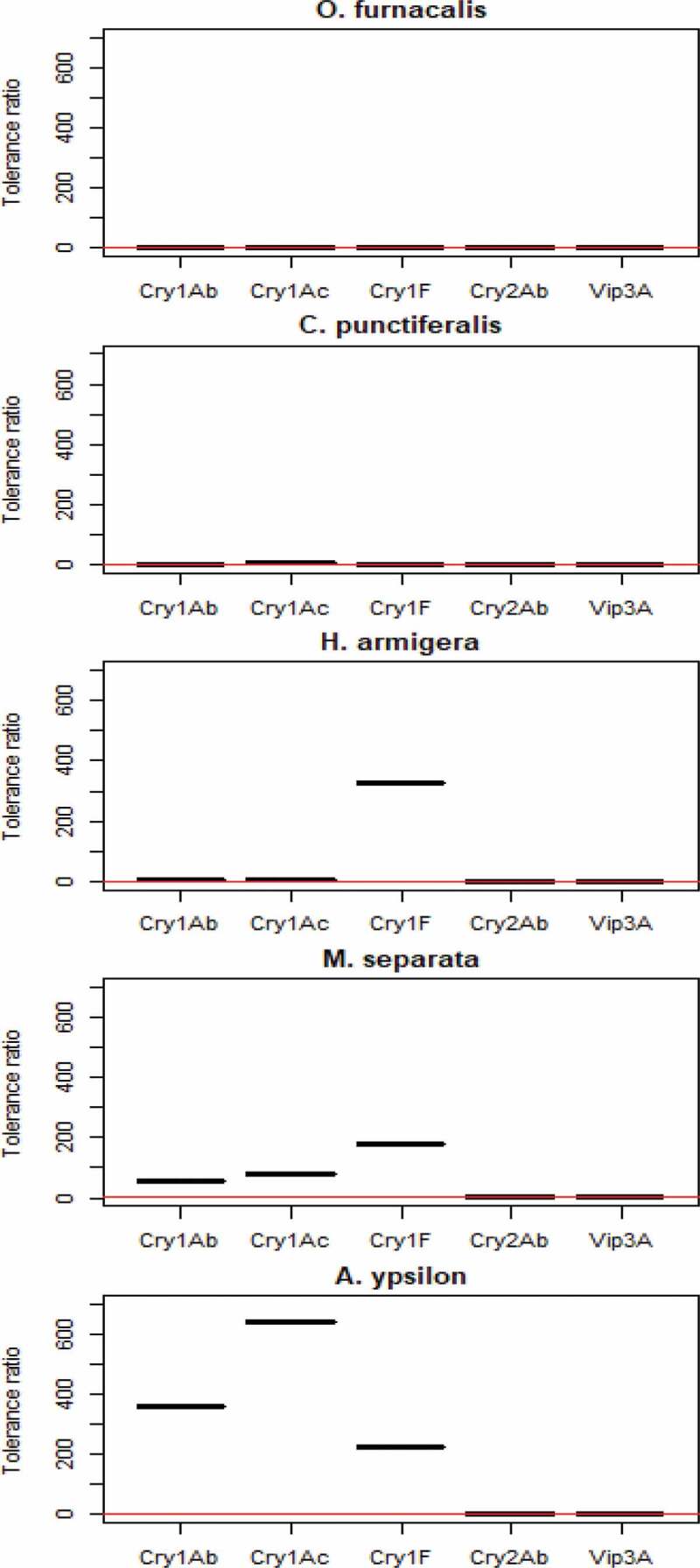
Tolerance ratio *C. punctiferalis, H. armigera, A. ypsilon and M. separata* relative to *O. furnacalis* for five Bt toxins (LC_50_ ratios of *C. punctiferalis, H. armigera, A. ypsilon* and *M. separata* relative to *O. furnacalis*)

The TR values show that the susceptibility profile of *C. punctiferalis* to different Bt toxins was similar to that of *O. furnacalis*. Both species were highly susceptible to Cry1Ab, Cry1Ac, and Cry1F, but relatively tolerant to Cry2Ab and to Vip3A (Fig. 1). In contrast, *H. armigera* was less susceptible to Cry1F, but similar compared with *O. furnacalis* and *C. punctiferalis* to other toxins. *A. ypsilon* and *M. separata* were less susceptible to the three tested Cry1 toxins, while *A. ypsilon* exhibited obvious tolerance to Cry1Ac, which is much different from *O. furnacalis* and *C. punctiferalis*.

## Discussion

Until now, no universal artificial diet is available for rearing different lepidopteran insect species, thus in our study artificial diets of different ingredients were used for different species. Other than this uncontrollable factor, the bioassay procedure and Bt proteins were handled the same as much as possible. Fortunately, all neonates showed a similar feeding behavior while being reared on artificial diets – initially scraped the available surface of the diet before tunneling. This behavior similarity ensured that different species took up approximately equal amount of Bt toxins, and enabled valid comparisons among the species to the best possible degree.

In this study, we quantified the differences of susceptibilities to different Bt toxins among the five main lepidopteran pests damaging summer corn in the HHHSCR of China. The results suggested that *O. furnacalis* and *C. punctiferalis* had very similar susceptibility patterns to the five Bt toxins, while *H. armigera, A. ypsilon*, and *M. separate* showed different susceptibility patterns. This information is critical for making decision on which type of Bt corn to be chosen for commercialization in the region. Such basic knowledge also is valuable for decision making in other regions or countries where the tested pests are distributed.

The first generation Bt maize varieties expressing only a single toxin (Cry1Ab or Cry1F) were commercially planted in 1996 in the USA to control the main lepidopteran pest, *O. nubilalis*, and other stalk-boring pests.^44^ Large area planting of these hybrid maize varieties have reduced the overall population of *O. nubilalis*.^45^ However, in the meantime a secondary pest *S. albicosta* has increased and become a major lepidopteran pest in some areas of the Corn Belt in the US and Canada due to low susceptibility of this species to most transgenic maize expressing Cry1Ab.^13,46–48^ In Europe, Bt corn expressing Cry1Ab toxin exhibits high efficacy against two primary lepidopteran borers, *S. nonagrioides* and *O. nubilalis*,^10,49^ but low efficacy against several secondary pests, such as *S. albicosta* and *M. unipuncta*,^50,51^ and similarly the latter two species are becoming more common in some European countries.^52^ To overcome this phenomenon, stacked Bt corn hybrids targeting multiple species expressing combinations of Cry1Ab, Cry1F, Cry1A.105, Cry2Ab and/or Vip3A became available more recently.^53^ Comparing with single-toxin hybrids, the stacked hybrids have advantages of reducing crop damage, improving control of individual pest species, broadening control spectrum, and reducing production of resistance phenotypes in a given population.^54,55^ These transgenic crops, including Cry1Ab+Cry1F, Cry1F+Cry2Ab, and Cry1Ab+Cry1F+Vip3A, can control or suppress a range of insect pests, including *H. zea* and *S. frugiperda*.^56^ Because pyramided and/or stacked Bt crops can enhance resistance management as well as pest control, they are expected to become more dominant in the future. The results of this study provide good guidance to seed developers for proper configuration of Bt toxins.

Developing proper Bt corn hybrid varieties for targeted production regions are essential to commercial utilization of transgenic Bt corn. *O. furnacalis, C. punctiferalis*, and *H. armigera* are three serious and common pests in the HHHSCR of China. *M. separate* and *A. ypsilon* are both of long distance migration pests, commonly occurring in this region. In view of these five major lepidopteran pests exhibited different degrees of susceptibility to different Bt toxins (Table 1, Fig. 1), planting transgenic corn expressing single Cry1Ab or Cry1Ac toxins might be highly efficacious against *O. furnacalis, C. punctiferalis, H. armigera, and M. separate*, but might not be effective against *A. ypsilon*. Similarly, planting Bt corn expressing single Cry1F toxin might be able to control *O. furnacalis* and *C. punctiferalis*, but might not control *H. armigera, M. separate* and *A. ypsilon*. Under these scenarios, planting corn cultivars that produced two or more Bt proteins, such as Cry1Ab (Cry1Ac) + Cry2Ab, Cry1Ab (Cry1Ac) + Vip3A, or Cry1F+ Cry2Ab (Vip3A) may be necessary in order to achieve satisfactory control. The finding of this study suggests that the first generation Bt corn expressing single Cry1 toxin should not be the first choice because of the potential risk of failure or less efficacious to control *H. armigera, A. ypsilon* or *M. separate*. The second-generation Bt corn expressing pyramided Cry1 and Cry2 toxins, or the third generation Bt corn expressing stacked Cry1, Cry2 and Vip3A toxins might produce better protection to corn and delay resistance evolution of target pests in the HHHSCR of China.

A brand new threat to corn in China is *S. frugiperda*, which invaded in 2019.^57,58^ Currently, this species has been detected in 21 provinces in SeHCR, SwHCR, and HHHSCR.^59^ *S. frugiperda* has been a severe problem in North America and Latin America where it attacks multiple crops and has developed resistance to Cry1F, Cry1Ab, and Cry2Ab corns.^60–65^ Fortunately, the invaded *S. frugiperda* population in China is highly susceptible to Cry1Ab, Cry1F, and Vip3A.^66^ Thus, planting corns of Cry1Ab (Cry1Ac) + Cry2Ab, Cry1Ab (Cry1Ac) + Vip3A, or Cry1F + Cry2Ab (Vip3A) in HHHSCR can also control this pest. However, since this invaded population might have originated from North America,^67^ it is possible that lower frequency resistance alleles might have been carried in already. Therefore, characterization of resistance allele frequency in this *S. frugiperda* population to different Bt proteins is necessary for resistance management in future.

In the present study, only laboratory bioassays with purified proteins were conducted to compare the pattern of susceptibility among the main lepidopteran pests. Without directly comparing the expression level of each protein in actual Bt corn events and plant tissue consumption by each pest, these results provide a starting point to determine what type of Bt corn is suitable for different corn production regions, and enable better preparation for transgenic Bt corn being authorized for commercial planting in China. In addition, Bt toxin baseline susceptibility data among main corn pest populations were documented. This broadens the database to model and assess the potential of resistance evolution to different Bt crops in combination with different pest species. Currently, the high-dose/refuge strategy is the most commonly recommended strategy for delaying resistance evolution. With this strategy, insects that feed on Bt maize are exposed to an extremely high dose of toxin, which makes insect resistance alleles functionally recessive. Since the toxicity of each protein varies depending on insect species, determination of efficacy of each Bt corn trait against all above lepidopteran pests to define a high dose should be systematically evaluated as we did in this study and in future.
